# *catena*-Poly[di-μ_1,1_-azido-(1,10-phenanthroline)cadmium(II)]

**DOI:** 10.1107/S1600536810019318

**Published:** 2010-06-09

**Authors:** Feng Chen, Fa-Kun Zheng, Guang-Ning Liu, Mei-Feng Wu, Guo-Cong Guo

**Affiliations:** a State Key Laboratory of Structural Chemistry, Fujian Institute of Research on the Structure of Matter, Chinese Academy of Sciences, Fuzhou, Fujian 350002, People’s Republic of China; b Graduate School, the Chinese Academy of Sciences, Beijing 100039, People’s Republic of China

## Abstract

The asymmetric unit of the title Cd^II^ compound, [Cd(N_3_)_2_(C_12_H_8_N_2_)]_*n*_, contains a Cd^II^ atom, located on a twofold axis passing through the middle of the phenanthroline mol­ecule, one azide ion and half of a 1,10-phenanthroline mol­ecule. The Cd^II^ atom exhibits a distorted octa­hedral coordin­ation including one chelating 1,10-phenanthroline ligand and four azide ligands. The crystal structure features chains along the *c* direction in which azide groups doubly bridge two adjacent Cd^II^ atoms in an end-on (EO) mode. Inter­chain π–π stacking inter­actions, with centroid–centroid separations of 3.408 (2) Å between the central aromatic rings of 1,10-phenanthroline mol­ecules, lead to a supra­molecular sheet parallel to the *bc* plane.

## Related literature

For the structures of related metal-azido compounds, see: Goher *et al.* (2008 ▶); Ribas *et al.* (1999 ▶); Liu *et al.* (2007 ▶); Cano *et al.* (2005 ▶); Abu-Youssef *et al.* (2000 ▶); Bose *et al.* (2004 ▶); Mautner *et al.* (2010 ▶); Meyer *et al.* (2005 ▶); Gao *et al.* (2004 ▶).
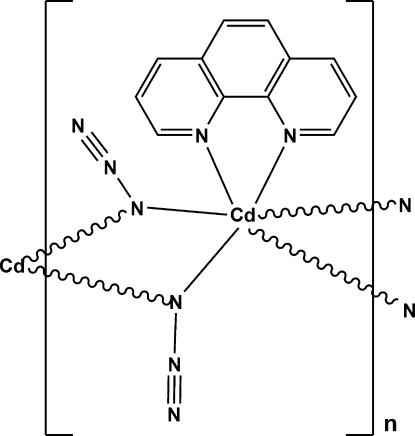


## Experimental

### 

#### Crystal data

[Cd(N_3_)_2_(C_12_H_8_N_2_)]
*M* *_r_* = 376.67
Monoclinic, 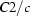

*a* = 19.4591 (17) Å
*b* = 10.2988 (6) Å
*c* = 6.8151 (6) Å
β = 106.033 (4)°
*V* = 1312.66 (18) Å^3^
*Z* = 4
Mo *K*α radiation
μ = 1.67 mm^−1^
*T* = 293 K
0.30 × 0.20 × 0.18 mm

#### Data collection

Rigaku Mercury CCD diffractometer
Absorption correction: multi-scan (*CrystalClear*; Rigaku, 2002 ▶) *T*_min_ = 0.774, *T*_max_ = 1.000
4185 measured reflections
1217 independent reflections
1133 reflections with *I* > 2σ(*I*)
*R*_int_ = 0.023

#### Refinement

*R*[*F* ^2^ > 2σ(*F* ^2^)] = 0.020
*wR*(*F* ^2^) = 0.056
*S* = 1.05
1217 reflections
96 parameters
H-atom parameters constrained
Δρ_max_ = 0.78 e Å^−3^
Δρ_min_ = −0.48 e Å^−3^

### 

Data collection: *CrystalClear* (Rigaku, 2002 ▶); cell refinement: *CrystalClear*; data reduction: *CrystalClear*; program(s) used to solve structure: *SHELXTL* (Sheldrick, 2008 ▶); program(s) used to refine structure: *SHELXTL*; molecular graphics: *SHELXTL*; software used to prepare material for publication: *SHELXTL*.

## Supplementary Material

Crystal structure: contains datablocks I, global. DOI: 10.1107/S1600536810019318/dn2567sup1.cif

Structure factors: contains datablocks I. DOI: 10.1107/S1600536810019318/dn2567Isup2.hkl

Additional supplementary materials:  crystallographic information; 3D view; checkCIF report

## Figures and Tables

**Fig. 1.**
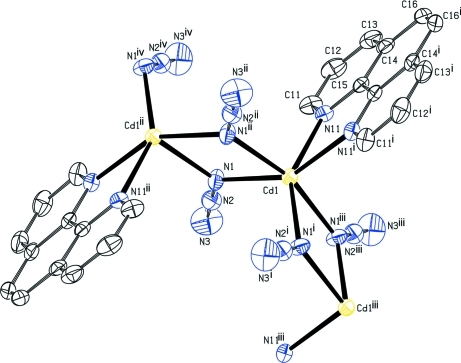
View of the title compound with the atom labeling scheme. Ellipsoids are drawn at the 30% probability level. H atoms have been omitted for clarity.[Symmetry codes: (i) -x+1, y, -z+1/2; (ii) -x+1, -y+2, -z; (iii) x, -y+2, z+1/2; (iv) x, -y+2, z-1/2].

**Fig. 2.**
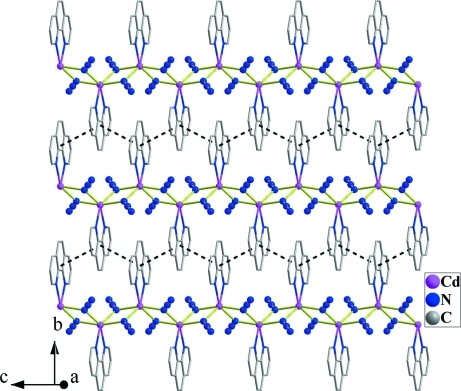
View of the 2-D layer structure of the title compound formed by 1-D CdII-azido chains linked through π-π stacking interactions (black dotted lines) between symetry related 1,10-phenanthroline molecules. Hydrogen atoms have been omitted for clarity.

## supplementary crystallographic information

### Comment

Many compounds with uncommon magnetic properties have been widely investigated by using azido ligand, resulting from its rich coordination fashions (Ribas *et al.*, 1999; Gao *et al.*, 2004). The azido ligand exhibits a variety of bridging modes such as bi-dentate end-on (EO) and end-to-end (EE) bridging fashions (Liu *et al.*, 2007; Cano *et al.*, 2005; Goher *et al.*, 2008; Mautner *et al.*, 2010). A number of compounds with various structures have been obtained by introducing auxiliary ligands to the metal-azido system (Abu-Youssef *et al.*, 2000; Bose *et al.*, 2004; Meyer *et al.*, 2005). The present example shows an infinite wavelike chain compound with 1,10-phenanthroline as an auxiliary ligand, [Cd(N_3_)_2_(C_12_H_8_N_2_)], in which azido ligand adopts the EO mode.

The asymmetric unit of the title compound contains half a Cd^II^ ion, one azido ion and half a 1,10-phenanthroline molecule (Fig. 1). The Cd^II^ ion exhibits a distorted octahedral geometry, coordinated by one chelating 1,10-phenanthroline ligand and four azido ligands with the end-on (EO) mode. The distances of Cd—N vary from 2.306 (2) to 2.411 (3) Å . The azido ligands doubly bridge neighbouring Cd^II^ centers in the EO fashion, yielding an infinite wave-like Cd^II^-azido chain along the *c* direction with the shortest Cd···Cd separation being 3.764 (3) Å.

The adjacent Cd^II^-azido chains are mediated by interchained π-π stacking interactions between the aromatic rings of 1,10-phenanthroline molecules, which arrange in the opposite direction alternatively. The centroid-to-centroid distance between the central rings of the phenanthroline is 3.408 (2)Å and the centroid-to-plane distance is 3.28 Å leading to a slippage of 0.936Å. This π-π stacking builts up a 2-D supramolecular layer parallel to the *bc* plane (Fig. 2).

### Experimental

A mixture of Cd(NO_3_)_2_.4H_2_O (0.308 g, 1.00 mmol), NaN_3_ (0.065 g, 1.00 mmol), Na(3-cba) (0.085 g, 0.50 mmol 3-Hcba = 3-cyanobenzoate acid), 1,10-phenanthroline (0.099 g, 0.50 mmol) and H_2_O (8 ml) was placed in a Teflon-lined stainless container, and then heated at 453 K for 2 days, after cooled to room temperature for 2 days. Pale-yellow prism-shaped crystals of the title compound were obtained. IR peaks (KBr, cm^-1^): 2053 s, 2037 s h, 1589 w, 1515 w, 1425 w, 1333 w, 1284 w, 846 m, 772 w, 727 m, 656 w. A strong band around 2053 cm^-1^ indicates the presence of the azido group.

### Refinement

Hydrogen atoms were allowed to ride on their respective parent atoms with C—H distances of 0.93 Å, and were included in the refinement with isotropic displacement parameters *U*_iso_(H) = 1.2*U*_eq_(C).

### Crystal data

**Table:**

[Cd(N_3_)_2_(C_12_H_8_N_2_)]	*F*(000) = 736
*M**_r_* = 376.67	*D*_x_ = 1.906 Mg m^−^^3^
Monoclinic, *C*2/*c*	Mo *K*α radiation, λ = 0.71073 Å
Hall symbol: -C 2yc	Cell parameters from 1622 reflections
*a* = 19.4591 (17) Å	θ = 2.3–27.5°
*b* = 10.2988 (6) Å	µ = 1.67 mm^−^^1^
*c* = 6.8151 (6) Å	*T* = 293 K
β = 106.033 (4)°	Prism, pale-yellow
*V* = 1312.66 (18) Å^3^	0.30 × 0.20 × 0.18 mm
*Z* = 4	

### Data collection

**Table:**

Rigaku Mercury CCD diffractometer	1217 independent reflections
Radiation source: fine-focus sealed tube	1133 reflections with *I* > 2σ(*I*)
graphite	*R*_int_ = 0.023
Detector resolution: 13.6612 pixels mm^-1^	θ_max_ = 25.5°, θ_min_ = 3.6°
CCD_Profile_fitting scans	*h* = −23→22
Absorption correction: multi-scan (*CrystalClear*; Rigaku, 2002)	*k* = −12→12
*T*_min_ = 0.774, *T*_max_ = 1.000	*l* = −8→8
4185 measured reflections	

### Refinement

**Table:**

Refinement on *F*^2^	Primary atom site location: structure-invariant direct methods
Least-squares matrix: full	Secondary atom site location: difference Fourier map
*R*[*F*^2^ > 2σ(*F*^2^)] = 0.020	Hydrogen site location: inferred from neighbouring sites
*wR*(*F*^2^) = 0.056	H-atom parameters constrained
*S* = 1.05	*w* = 1/[σ^2^(*F*_o_^2^) + (0.0373*P*)^2^ + 0.2202*P*] where *P* = (*F*_o_^2^ + 2*F*_c_^2^)/3
1217 reflections	(Δ/σ)_max_ = 0.001
96 parameters	Δρ_max_ = 0.78 e Å^−^^3^
0 restraints	Δρ_min_ = −0.48 e Å^−^^3^

### Special details

**Table:**

Geometry. All esds (except the esd in the dihedral angle between two l.s. planes) are estimated using the full covariance matrix. The cell esds are taken into account individually in the estimation of esds in distances, angles and torsion angles; correlations between esds in cell parameters are only used when they are defined by crystal symmetry. An approximate (isotropic) treatment of cell esds is used for estimating esds involving l.s. planes.
Refinement. Refinement of *F*^2^ against ALL reflections. The weighted *R*-factor wR and goodness of fit *S* are based on *F*^2^, conventional *R*-factors *R* are based on *F*, with *F* set to zero for negative *F*^2^. The threshold expression of *F*^2^ > σ(*F*^2^) is used only for calculating *R*-factors(gt) etc. and is not relevant to the choice of reflections for refinement. *R*-factors based on *F*^2^ are statistically about twice as large as those based on *F*, and *R*- factors based on ALL data will be even larger.

### Fractional atomic coordinates and isotropic or equivalent isotropic displacement parameters (Å^2^)

**Table:**

	*x*	*y*	*z*	*U*_iso_*/*U*_eq_	
Cd1	0.5000	0.922344 (19)	0.2500	0.03485 (12)	
N1	0.43055 (13)	1.0473 (2)	−0.0103 (3)	0.0446 (5)	
N2	0.37375 (13)	1.0889 (2)	−0.0144 (4)	0.0459 (6)	
N3	0.31894 (16)	1.1315 (4)	−0.0167 (6)	0.0898 (10)	
N11	0.43063 (10)	0.73525 (19)	0.1345 (3)	0.0367 (4)	
C11	0.36250 (14)	0.7349 (3)	0.0214 (4)	0.0488 (6)	
H11A	0.3404	0.8140	−0.0213	0.059*	
C12	0.32340 (16)	0.6220 (4)	−0.0352 (5)	0.0587 (8)	
H12A	0.2758	0.6260	−0.1114	0.070*	
C13	0.35523 (17)	0.5055 (3)	0.0218 (4)	0.0551 (8)	
H13A	0.3293	0.4291	−0.0144	0.066*	
C14	0.42742 (16)	0.5003 (2)	0.1357 (4)	0.0447 (6)	
C15	0.46270 (13)	0.6200 (2)	0.1908 (3)	0.0344 (5)	
C16	0.46560 (18)	0.3819 (3)	0.1969 (4)	0.0552 (8)	
H16A	0.4420	0.3032	0.1619	0.066*	

### Atomic displacement parameters (Å^2^)

**Table:**

	*U*^11^	*U*^22^	*U*^33^	*U*^12^	*U*^13^	*U*^23^
Cd1	0.03961 (17)	0.03359 (16)	0.02969 (16)	0.000	0.00680 (11)	0.000
N1	0.0461 (13)	0.0517 (11)	0.0384 (12)	0.0101 (11)	0.0156 (10)	0.0139 (10)
N2	0.0417 (14)	0.0477 (13)	0.0439 (13)	0.0009 (10)	0.0047 (10)	−0.0005 (9)
N3	0.0428 (16)	0.116 (3)	0.105 (3)	0.0208 (18)	0.0108 (16)	−0.008 (2)
N11	0.0368 (10)	0.0410 (11)	0.0332 (10)	0.0009 (9)	0.0109 (8)	−0.0043 (8)
C11	0.0405 (13)	0.0601 (17)	0.0449 (14)	0.0015 (13)	0.0099 (11)	−0.0113 (13)
C12	0.0430 (15)	0.084 (2)	0.0481 (16)	−0.0126 (16)	0.0112 (13)	−0.0179 (16)
C13	0.0651 (19)	0.0630 (18)	0.0434 (15)	−0.0282 (16)	0.0257 (14)	−0.0187 (13)
C14	0.0678 (18)	0.0436 (14)	0.0314 (12)	−0.0137 (12)	0.0282 (13)	−0.0088 (10)
C15	0.0436 (13)	0.0385 (11)	0.0241 (11)	−0.0021 (11)	0.0146 (10)	−0.0024 (9)
C16	0.099 (2)	0.0354 (11)	0.0409 (16)	−0.0135 (14)	0.0363 (15)	−0.0077 (11)

### Geometric parameters (Å, °)

**Table:**

Cd1—N1^i^	2.303 (2)	C11—C12	1.385 (5)
Cd1—N1	2.303 (2)	C11—H11A	0.9300
Cd1—N11	2.3596 (19)	C12—C13	1.357 (5)
Cd1—N11^i^	2.3596 (19)	C12—H12A	0.9300
Cd1—N1^ii^	2.411 (2)	C13—C14	1.406 (4)
Cd1—N1^iii^	2.411 (2)	C13—H13A	0.9300
N1—N2	1.179 (3)	C14—C15	1.410 (4)
N1—Cd1^ii^	2.411 (2)	C14—C16	1.429 (4)
N2—N3	1.149 (4)	C15—C15^i^	1.453 (5)
N11—C11	1.337 (3)	C16—C16^i^	1.335 (7)
N11—C15	1.347 (3)	C16—H16A	0.9300
			
N1^i^—Cd1—N1	112.07 (12)	C11—N11—Cd1	125.41 (18)
N1^i^—Cd1—N11	150.83 (8)	C15—N11—Cd1	116.58 (15)
N1—Cd1—N11	92.25 (8)	N11—C11—C12	122.9 (3)
N1^i^—Cd1—N11^i^	92.25 (8)	N11—C11—H11A	118.5
N1—Cd1—N11^i^	150.83 (8)	C12—C11—H11A	118.5
N11—Cd1—N11^i^	70.51 (9)	C13—C12—C11	119.4 (3)
N1^i^—Cd1—N1^ii^	97.46 (8)	C13—C12—H12A	120.3
N1—Cd1—N1^ii^	74.05 (9)	C11—C12—H12A	120.3
N11—Cd1—N1^ii^	104.83 (8)	C12—C13—C14	119.9 (3)
N11^i^—Cd1—N1^ii^	87.47 (7)	C12—C13—H13A	120.0
N1^i^—Cd1—N1^iii^	74.05 (9)	C14—C13—H13A	120.0
N1—Cd1—N1^iii^	97.46 (8)	C13—C14—C15	116.9 (3)
N11—Cd1—N1^iii^	87.47 (7)	C13—C14—C16	123.6 (3)
N11^i^—Cd1—N1^iii^	104.83 (8)	C15—C14—C16	119.5 (3)
N1^ii^—Cd1—N1^iii^	165.09 (11)	N11—C15—C14	122.8 (2)
N2—N1—Cd1	124.66 (19)	N11—C15—C15^i^	118.13 (13)
N2—N1—Cd1^ii^	129.35 (18)	C14—C15—C15^i^	119.09 (16)
Cd1—N1—Cd1^ii^	105.95 (9)	C16^i^—C16—C14	121.41 (17)
N3—N2—N1	178.8 (3)	C16^i^—C16—H16A	119.3
C11—N11—C15	118.0 (2)	C14—C16—H16A	119.3

Symmetry codes: (i) −*x*+1, *y*, −*z*+1/2; (ii) −*x*+1, −*y*+2, −*z*; (iii) *x*, −*y*+2, *z*+1/2.
